# Instrumented Four Square Step Test in Adults with Transfemoral Amputation: Test-Retest Reliability and Discriminant Validity between Two Types of Microprocessor Knees

**DOI:** 10.3390/s20174782

**Published:** 2020-08-24

**Authors:** Arnaud Gouelle, Michael Jason Highsmith

**Affiliations:** 1 ProtoKinetics, Havertown, PA 19083, USA; 2 Laboratory «Performance, Santé, Métrologie, Société (PSMS)», UFR STAPS (University of Sport Sciences), 51100 Reims, France; 3 School of Physical Therapy & Rehabilitation Sciences, Morsani College of Medicine, University of South Florida, Tampa, FL 33612, USA; mhighsmi@usf.edu

**Keywords:** FSST, transfemoral amputation, prosthesis, microprocessor knee, instrumentation

## Abstract

Technology-based outcomes have recently been proposed to complement the standard Four Square Step Test (FSST) by providing a decomposition of the sequences and information about the stepping pattern. A test-retest study and a randomized crossover design have been used to determine immediate test-retest reliability and to assess discriminant validity, in persons with a unilateral transfemoral amputation, for the parameters computed by an instrumented version of the Four Square Step Test. Twenty adults, independent and unlimited community ambulators, with a unilateral transfemoral amputation, performed two Four Square Step Tests on a pressure mat first with a microprocessor knee, then, a few weeks later with another one. One of these prosthetic knees was acknowledged to be superior and to provide functional improvement. Test-retest, intraclass correlation coefficients and minimal detectable change at 95% confidence level were calculated for each variable. Paired samples t-tests were then used to identify differences between the two microprocessor knee systems. The test-retest reliability of most outcome measures was good to excellent. Few variables showed a systematic difference and a trend to improve between test 1 and test 2. When comparing both microprocessor knees, significant differences in the expected direction were observed, with interpretation in accordance with a functional improvement. Importantly, we highlighted that various strategies to improve the performance in the test might complexify the interpretation of the most detailed measurement. The instrumented Four Square Step test provides reliable measures with satisfactory test-retest reliability and discriminant validity in persons with unilateral transfemoral amputation.

## 1. Introduction

Restoring a person’s functional abilities is a primary goal in individuals with a lower extremity amputation. That is even if semantically ‘restoring’ might be an inappropriate term, when the individual has to learn how to move and maintain balance with sensory loss, limited degrees of freedom and less control capabilities than he/she was used to prior to amputation. Managing physical rehabilitation and prosthetic fitting is also an interesting, but complex, challenge for the clinician, as so many factors influence the prosthetic design and the individual’s response, such as the physical characteristics of the residual limb, range of motion at proximal joints, muscle strength, activity level, hobbies, patient goals, age, gender, living situation, time since amputation, previous prostheses, comorbidities and others. Walking ability and static balance assessments are widely used in clinical settings to determine limitations in persons with a prosthetic limb. Individuals with unilateral lower-limb amputation have altered static postural sway and reduced capabilities for maintaining balance [1], and, when considering walking, many gait parameters differ from a standard healthy gait pattern [2]. These subjects rely particularly more on their sound limb to compensate for the deficiencies associated with the prosthesis, which contributes to intact side overload and leads to spatial, temporal, and loading asymmetries [1,2]. For example, a longer sound side stance can be ascribed to the greater ability of the sound leg to advance the step and maintain balance [3,4]. Over time, the increased loading period and compensatory motions may explain the development of complications in the sound limb because significant differences between the intact and amputated limb may lead to strain and initiate or accelerate degenerative change [5]. Therefore, the inter-limb symmetry, is commonly regarded as an important consideration for rehabilitation of amputee gait [6,7]. Nevertheless, static balance assessments and standard gait analysis provides limited insight to everyday mobility, as they are constrained to situations of minimal environmental challenge. If they give valuable information during the rehabilitation after amputation, once the person has reached a certain level of functionality and confidence with the prosthesis, it can be difficult to gauge if an intervention has a significant effect, such as when effective testing of different instrumented prosthetic devices is required. Therefore, it is important to examine outcome measures containing tasks that challenge dynamic balance.

The Four Square Step Test is one such suitable test which involves human stepping performance. Dite and Temple [8] developed the Four Square Step Test to examine the ability to step over small objects and to change direction within a clinical setting. The Four Square Step Test consists of a timed measure that requires individuals to step over canes placed in a crosswise pattern on the floor, thereby creating four quadrants [9]. The subject starts in one square and steps successively through the squares, first in a clockwise then back in a counterclockwise direction. The Four Square Step Test has been validated in several populations, including faller older adults [8], people with vestibular disorders [10], stroke [11], multiple sclerosis [12], Parkinson’s disease [13,14], and individuals with lower-limb amputation [15]. While healthy active adults aged younger than 30 years can complete the Four Square Step Test in under 6 s, 50 to 65-year old persons in 7.49 s, and healthy people aged 65 to 80 years are able to perform it in 10 s or less [16], a range of cut-off scores for prediction of multiple fallers was identified from as low as 9.68 s in those with Parkinson’s disease [13] up to 24 s following unilateral transtibial amputation [15]. The different results for concurrent, construct, and predictive validities, as well as test-retest reliability, suggest that the Four Square Step Test may be an effective and valid tool to measure dynamic balance [17]. However, when the Four Square Step Test requires that the person performs rapid stepping in different directions, with both left/right and forward/backward directed movement as well as alternating limb support, the only outcome taken from the test is the total duration in seconds and many different movement strategies can lead to an equivalent total duration to perform the test (see Video S1 provided as Supplementary Material). More in-depth analyses would complement the assessment to gain a more objective and detailed view of functionality, especially for the persons with a unilateral transfemoral amputation, who demonstrate inherent functional asymmetry because of the missing biological knee joint.

Pressure-sensing mats have this capacity to record sensors’ activity data which allow extraction of additional information about the performance. From the pressure data captured by an electronic Zeno™ walkway when the person is performing a Four Square Step Test on it, a decomposition of the performance has been proposed and provides outcomes related to the way the person does the successive transfers. There are reasonable grounds to consider that this iFSST could help to evaluate the functional effect of an instrumented knee prosthetic devices on human movement performance. To date, the instrumented Four Square Step Test has demonstrated excellent reliability to manual timing in participants with Parkinson’s disease [18], but there is neither report about the reliability of the different parameters given by this instrumented Four Square Step Test, nor previous finding investigating functional differences between two instrumented knee prostheses. Therefore, the purpose of this study was two-fold: (1) to establish estimates of immediate test-retest reliability and minimal detectable change values of the instrumented Four Square Step Test (iFSST) parameters in persons with a unilateral transfemoral amputation; then (2) to compare outcomes from the same individuals performing with two different types of microprocessor knee systems. Based on the technical aspects and previous articles [19,20], we expected to identify some improvements with one specific model.

## 2. Materials and Methods

The study was approved by the University of South Florida’s Institutional Review Board and listed in a federal clinical trial registry. All subjects gave informed consent prior to participation in the study.

### 2.1. Participants

A randomized crossover design has been set up for a wider study [19], where people with unilateral transfemoral amputation were tested with two different microprocessor knee systems (named the C-Leg and the Genium systems; Ottobock Healthcare, Duderstadt, Germany), in random order separated by an accommodation period of >2 weeks to <3 months. The C-Leg receives sensor input at 50 Hz and has an 8 MHz microprocessor in addition to a knee angle sensor to determine sagittal knee position and an ankle moment sensor to determine sagittal moments of the ankle. Beyond these two sensors, four additional sensors are incorporated into the Genium system. The four additional sensors are an axial pylon load sensor, a sagittal knee moment sensor, a biaxial accelerometer, and a gyroscope. Additionally, the Genium receives sensor input at an increased 100 Hz and has an 18.6 MHz processing speed to facilitate increased responsiveness to changes in cadence, walking slope, posture, and movement cessation [20]. Protocol details including randomization and interventions, as well as fitting and accommodation periods are described elsewhere [19]. Subjects had to have unilateral transfemoral amputation from any etiology and not have impairments that adversely impacted their gait beyond their amputations (e.g., cardiopulmonary, orthopedic impairments). Additionally, they had to be C-Leg users for ≥1 year prior to enrollment. Prosthetic sockets and suspension systems were not changed for the experiment’s duration.

### 2.2. Procedure

**Figure 1:**
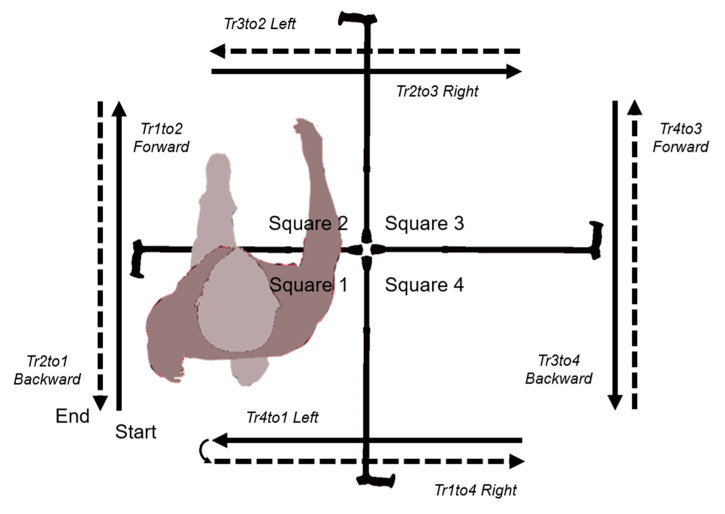
Sequence scheme for the Four Square Step Test.

The procedure was the same as for a standard Four Square Step Test [8]. First, the person stood in square 1 in a comfortable position. A start command was given, then the subject sequentially stepped over four walking canes placed in a cross configuration on the ground, first in a clockwise direction, then reverse in a counterclockwise direction (Figure 1). Subjects were instructed to try to complete the test as fast as possible without touching the sticks. They were asked to contact with both feet in each square and, if possible, face forward during the entire sequence. The rater provided a demonstration and asked the person to perform a non-scored practice trial prior to recording performance. Two trials were then conducted. If a subject’s foot touched a cane, the trial was stopped and repeated until two successful trials were completed. All the persons performed the test with one type of microprocessor knee system at a first session, then with the second type at a second session after >2 weeks to <3 months.

### 2.3. Instrumentation

The Four Square Step Test was performed on a 1.22 m by 1.22 m area of an electronic mat acquiring data at 120 Hz (Zeno Walkway; ProtoKinetics, Haverton, PA, USA) and recorded with the instrument’s software (PKMAS). The system has been validated both for spatiotemporal parameters and center of pressure [21,22] and has previously been used to analyze the Timed-Up-and-Go in subjects with unilateral lower-limb amputation [23]. Algorithms implemented into the software automatically processed the iFSST, identified the four squares and the successive left/right footprints, then provides spatiotemporal aspects of the footsteps and information about the stepping pattern.

### 2.4. Data Processing

#### 2.4.1. Timing

For a standard, hand-scored Four Square Step Test [8], the stopwatch starts when the first foot touches the floor in square 2. Once the command has been given, the operator has a short time to anticipate placement of the starting foot into square 2 which represents a more reliable visual cue. The use of a pressure mat allows accurate identification of both anticipatory postural adjustments during step initiation and contact events. For the instrumented Four Square Step Test, the performance is considered between the instant when the starting foot left square 1 until the contact of the last foot in square 1. It allows to be close to the original Four Square Step Test, while integrating the first swing phase and being consistent with the event considered as the end of the test.

#### 2.4.2. Temporal Decomposition

The instrumented Four Square Step Test trial has been parsed into multiple time segments. A subject is always in one of two time-segment types which are non-overlapping and contain no time gaps: either in a square or transitioning between two squares. For example, the transition from square 1 to square 2 (hereafter noted as Tr1to2) is represented by the time segment starting at the end of the last double support where both feet are in square 1 to the start of the first double support where both feet are in square 2. The subject is then in square 2 during the time segment which begins at the start of the first double support and ends with the last double support in this square. Since the person must step two times through each square, the periods of time spent in one square is indicated by the number of the square followed by one 1 or 2, for the first and second pass in this square. Therefore, a complete instrumented Four Square Step Test is comprised of the following sequence: Tr1to2, Sq2–1, Tr2to3, Sq3–1, Tr3to4, Sq4–1, Tr4to1, Sq1–2, Tr1to4, Sq4–2, Tr4to3, Sq3–2, Tr3to2, Sq2–2, Tr2to1. For normalization purpose, the durations have been expressed as a percentage of the total duration of the instrumented Four Square Step Test.

#### 2.4.3. Adaptation to Consider the Prosthetic Side

Given the asymmetric nature of the studied sample, the duration of each phase can be more limb-dependent rather than sequence dependent. For this reason, we looked at the side of the prosthesis to provide the time (in seconds) of the first limb transfer (i.e., its swing phase) in accordance with the direction of the movement. For example, for a person with a right amputation, each transfer toward the right was initiated by the prosthetic limb and each transfer toward the left was initiated by the sound limb. The swing phase duration for the two transfers toward the right (Tr2to3 and Tr1to4) have been averaged, as well as the duration for the two transfers toward the left (Tr4to1 and Tr3to2). Finally, we computed a temporal symmetry ratio as the duration of transfer toward the prosthetic side (i.e., the prosthetic limb is the first to transfer) divided by the duration of transfer toward the sound side (i.e., the sound limb is the first to transfer). The same procedure has been followed with the step width (i.e., the distance in centimeters between the last position of the foot in one square and the first position of the foot in the next one). Therefore, a spatial symmetry ratio was computed as the distance achieved during the transfer toward the prosthetic limb side (i.e., the prosthetic limb is the first to transfer) divided by the distance achieved during the transfer toward the sound side (i.e., the sound limb is the first to transfer).

#### 2.4.4. Stepping Pattern

**Figure 2:**
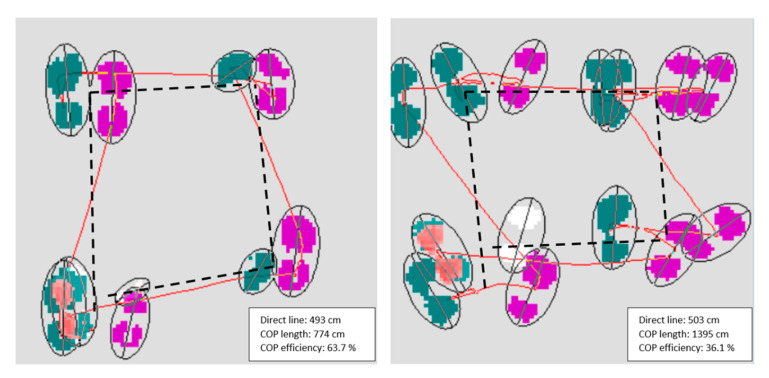
Illustration of the distances used for the computation of the global center of pressure (COP) efficiency, for a good (**left**) and a bad ratio (**right**). The red line represents the COP pathway and the black line represents the distances between the middle of each couple of footprints in successive squares. The displays only show the first four couples of footprints.

The stepping pattern has been assessed through three iFSST variables provided by the software: the number of extra steps taken to perform the iFSST; the number of changes of the support limb side; the pathway’s efficiency of the center of pressure (COP; variables are defined below). For an optimal pattern, eighteen footprints should be observed, two in each square, including the standing position in square 1 and the final standing position in square 1. Any steps over this limit is then considered as an extra step. The changes in the support limb indicate how many times the subject transfers weight from one side to the other. This measurement is based on the pressure levels which allows determination when a change of support has occurred. For the efficiency, the position of the footprints in successive squares are considered to derive a distance which serves as a reference to normalize the center of pressure pathway. In detail, the central position between left/right footprints is determined in each square and the total length between these successive positions through the iFSST is calculated (Figure 2, black ‘direct line’). Then, a ratio is computed between the full length of the center of pressure pathway and the ’direct line’ (Figure 2). This is a mathematical conceptualization of what would be the most direct pathway of the center of pressure, not what could be achieved in reality; however, this makes it possible to analyze the pathway of the center of pressure by making it independent of the leg length and/or of the step length/width.

### 2.5. Statistics

All statistical analyses were performed using SPSS version 25 (IBM Corp., Armonk, NY, USA) and differences were considered significant if *p*-values were less than 0.05. An a priori power analysis indicated that a sample size of 18 was adequate to establish that a detected reliability coefficient above 0.80 (good reliability) was significantly different from a reliability coefficient below 0.5 (cut-off value for poor reliability) with a power of 0.80 and an alpha of 0.05 [24].

#### 2.5.1. Reliability Analysis

Each of the twenty participants had two iFSST trials recorded during a first session with one random type of microprocessor knee system (MKS1–FSST1 and MKS1–FSST2) and two iFSST trials recorded during a second session a few weeks later with the other type of microprocessor knee system (MKS2–FSST1 and MKS2–FSST2). All coupled trials (i.e., iFSST1 and iFSST2 of a same session) were used to address immediate test-retest reliability. Twenty individuals with two sessions lead to a total of forty paired trials. The normality of continuous variables’ distribution was first evaluated by Kolmogorov Smirnov’s test and the homogeneity of variance by Levene’s test. Afterwards, intraclass correlation for test-retest absolute agreement for a single rater (intraclass correlation coefficient, ICC2,1) were calculated, as well as the 95% confidence interval, standard error of measurement and minimal detectable change at a 95% confidence level [25]. Test-retest reliability was interpreted as “poor” if the intraclass correlation coefficient value was below 0.5, “moderate” if from 0.5 to 0.75, “good” if from 0.75 to 0.9, and excellent if above 0.90 [26]. Systematic differences were identified using a paired samples t-test.

#### 2.5.2. Comparison between the Microprocessor Knee Systems

The best trial (i.e., fastest time to complete the iFSST) for each session has been kept for this analysis. For example, if at the first session, the first iFSST (MKS1–FSST1) was completed in less time than the second one (MKS1–FSST2), the trial MKS1–FSST1 was kept. The same process was then followed to determine the fastest trial between the trials at the second session (MKS2–FSST1 or MKS2–FSST2). At this time, we attained twenty couples of variables, each person having a best trial for session 1 and a best trial for session 2. Then, to avoid any bias due to the foot initiating the test, we removed couples of paired variables when the person did not start with the same limb in both two trials. Paired samples t-tests were then used to identify differences between the two prosthetics limbs.

## 3. Results

Twenty adults with unilateral transfemoral amputation (Table 1) consented and completed all trials for the iFSST. Most subjects were male (80%) with a mean (SD) age of 46.5 years (14.2) and body mass index of 26.4 kg/m^2^ (4.2). All subjects were independent, unlimited community ambulators. Mean time since amputation was 17.7 years (15.6), and amputation etiology was predominantly traumatic (68%) followed by malignancy (21%) and peripheral vascular disease (11%). Seven subjects had left-sided amputations (35%).

### 3.1. Test-Retest Reliability

Mean (SD) for Test 1 and Test 2 are given in Table 2 and Table 3, with intraclass correlation coefficients, standard error of measurement and minimal detectable change at 95% confidence level. The absolute reliability of most variables (24 on 30) was good to excellent, and moderate for the six others. Particularly, when considering the most global parameters, such as the iFSST duration, the time spent on the sound or the prosthetics side, and the stepping pattern variables (i.e., extra steps, changes in main support, pathway’s efficiency of the center of pressure), these variables demonstrated excellent reliability (intraclass correlation coefficients ranged from 0.90 [0.80–0.95] to 0.97 [0.95–0.99]). Parameters with moderate reliability were relative durations in transitions Tr3to4, Tr4to1 and in squares Sq1–2 and Sq3–2 (intraclass correlation coefficients ranged from 0.66 [0.36–0.82] to 0.74 [0.50–0.87]). Duration on the sound side and extra steps, like four other variables, demonstrated small yet statistically significant change in values from Test 1 to Test 2. All other variables had non-significant paired t-tests, reflecting the absence of systematic difference between tests (Table 2). For the additional variables that we computed to address the directionality of the movement during the iFSST in accordance with the prosthetics side, reliability was good for the step width during lateral transitions toward the sound side, was moderate for the swing phase duration during transitions toward the sound side, the swing phase duration and the step width during transitions toward the prosthetic side. Reliability for the asymmetry ratios, between variables for the transitions toward the prosthetic side and transitions toward the sound limb, were poor, however without significant difference between Test 1 and Test 2.

### 3.2. Differences between the Microprocessor Knee Systems

Once we considered the best trial, 16 of the 20 persons of our sample had started with the same foot in both conditions and thus their data were kept for further analysis (Table 4 and Table 5). The duration of the iFSST was not significantly different between the two knee systems, with means [95% Confidence Interval] of 11.1 s [10.1–12.1] and 10.4 s [8.9–11.9] for CLeg and Genium prosthesis, respectively. Sound side duration demonstrated a decrease with the Genium when expressed in seconds as well as in percentage of the whole instrumented Four Square Step Test, not the prosthetics side duration. The stepping pattern was different for the number of extra steps and changes of main support, with less in the Genium condition (respectively 1.4 ± 1.6 and 15.8 ± 3.7) than with the CLeg (2.3 ± 1.7 and 17.3 ± 2.0). When considering the decomposition into transitions and squares, relative duration in transitions Tr1to2, Tr4to1, Tr4to3 and Tr3to2 was higher with the Genium, while relative duration in squares Sq4–1 and Sq1–2 was lower. Ratios between time spent in transitions and squares consequently increased with the Genium microprocessor knee system. Regarding the variables which considered directionality and side of the prosthetics limb, no differences were identified between the knee systems.

## 4. Discussion

### 4.1. Test-Retest Reliability

This study provided evidence related to immediate test-retest reliability and minimal detectable change values of the outcomes from an instrumented Four Square Step Test in persons with unilateral transfemoral amputation. Our results suggest on one hand, that most measures had “good” to “excellent” test-retest reliability, especially the stepping pattern variables, reflecting the ability of the protocol to provide consistent test-retest measurements of performance, while on the other hand, that the various possible ways to perform a FSST trial induce complexity when interpreting results.

Established minimal detectable change values can help identify a true change in measured performance that is beyond random variations or spurious findings [25]. The minimal detectable change at the 95% level for the iFSST total duration was 1.5 s, which seems to constitute a relatively small difference to distinguish what can be a true improvement or decrease in functional performance between two sessions for the same individual. For example, for an assessment such as the Timed-Up-and-Go, for which in a previous study of persons with a lower-limb amputation [27] the total duration was close to the duration of the iFSST in our study, the minimal detectable change at 90% level was 3.3 s. Based on the current results, less than 2.5% of variation is also needed to see a change in the relative durations, referring to the decomposition of the test into periods of transition between squares or within a square, except for Sq1–2. This square is the only one which implies two successive transitional movements in the frontal plane and is the location where the direction changes from the clockwise part of the iFSST to the counterclockwise portion. In other words, a transition leftward is directly followed by a transition rightward. For a person with a left amputation, it implies to rely for a longer time on the prosthetic side or, if not possible, to make extra steps or to unload the prosthetic limb to the sound limb to ensure balance. Therefore, this period of the iFSST can constitute a difficult part for an individual, but it also represents a period which can be improved between two consecutive tests. An analysis focused on the directionality of the movement and the prosthetic side also highlighted limb-dependent differences depending on whether or not the lateral movement is toward the prosthetic side (i.e., this side is first in swing phase then has to support the weight during the time that the sound limb is transferring) or the sound side. The swing phase duration and the step width were shorter for the transition of the limb toward the prosthetic side than the sound side. In other words, the prosthetic limb was moved less distance and was brought to the floor faster than the sound limb, in what might be interpreted as a higher dependency upon the sound limb.

Besides, few variables demonstrated systematic difference between test and retest. The duration of the time spent on the sound limb decreased especially, while it did not change for the prosthetic side. Fewer extra steps were also required to perform Test 2. Together, these differences were in favor of an improvement at the second assessment, because the more challenging the test is for balance, the more control and regulation of dynamic instabilities are required by the intact limb. To our knowledge, the test-retest of the standard Four Square Step Test has not been specified in persons with transfemoral amputation. However, in a study in persons with Parkinson’s disease, McKee and Hackney [14] measured test-retest during three different Four Square Step Test trials and found better intraclass correlation coefficients between trials 2 and 3 than between 1 and 2. Their mean durations showed an improvement though out the three trials. Due to the transitional nature of the test and the multiple foot placement sequences which are possible, it is improbable that a subject can plan the optimal sequence to realize, but it seems that more repetitions induces a better performance, as the person becomes more confident with the test. If we let the subject perform one practice trial before recording the two tests of the iFSST, we were not certain how learning effect would affect subsequent analysis of the differences between the two microprocessor knee systems and, for this reason, only the best iFSST performance with each prothesis was then kept for comparison. Also, the first transition of the test, i.e., Tr1to2, were statistically different between Test 1 and Test 2, which might be explained by the starting limb, which is not constrained. As for gait initiation in persons with a lower-limb amputation, if the prosthetic limb constitutes the preferred lead limb in 71% of the persons with transfemoral amputation [28], which matches with our current observations, the iFSST was also initiated in some cases with the sound limb.

### 4.2. Difference between Microprocessor Knee Systems

Based on the technical aspects and previous articles [19,20], we expected to identify some improvements with the Genium microprocessor knee system. Indeed, this system incorporates additional sensors to determine axial load, slope, and direction of travel as well as knee moments. These technical improvements have already shown clear trends of functional improvement identifiable between the C-Leg and Genium systems, whereby the Genium system reduces functional and activity impairment in activities of daily living, in the direction of nonamputee controls [20]. Our current results trended in the same direction with multiple difference between the C-Leg and the Genium knee, with Genium use having improved outcomes. This underscores the interest of the iFSST as an option to characterize mobility changes in persons with unilateral transfemoral amputation, in cases where the total duration of the test, usually the only outcome measure when performing a standard FSST, failed to discriminate between conditions. Better performances are achieved when subjects do not take too much time between each transfer. This duration in a square relies in high proportion on the stepping pattern, with the number of extra steps and changes in main support. In terms of outcomes, a better performance is then demonstrated by an increase in relative duration of the transitions and, on the contrary, by a decrease in relative duration in the squares. We identified that the relative duration on the sound limb decreased with the Genium, possibly due to less need for regulation from the sound side, where more muscles and degrees of freedom can be involved in case of balance perturbation. Variables which characterize the stepping pattern, both how many steps the person takes and how many times the weight is toppled from one limb to the other, decreased significantly with the Genium. The difference in the mean value of each variable with the C-Leg and the Genium had not reached the level of the minimal detectable change at 95% level that we determined in the first part of this work. However, an analysis of the individual results of the persons with unilateral transfemoral amputation indicates this level was overpassed for some persons, but not always for the same variables. In this view, the iFSST might show the highest clinical utility by giving indication to highlight the deficits and movements which are problems for the person. Side steps and backwards steps are both part of daily-life activities, like moving laundry from a washer to a dryer or to step back off the road if a car were approaching, but a person with unilateral transfemoral amputation might need more strength or adjustment in one of these directions, quantifiable by variables of the iFSST related to lateral or backward transitions.

### 4.3. What Can We Learn from iFSST

A test such as the iFSST is particularly suitable to effectively assess dynamic balance abilities because the task involves components of balance which are not incorporated in standard clinical measurements. The ability to perform rapid stepping in different directions and for the foot to clear the floor is essential in daily life. The measure of human stepping performance is undoubtedly valuable to monitor neuro-motor dysfunction. In persons with unilateral amputation, a well-fitted and properly adjusted prosthetic limb is critical for mobility. However, a sub-optimal prosthetic fitting or adjustment may not necessarily yield a significant change in total Four Square Step Test duration. The additional variables demonstrated in this work and available with simultaneous use of a pressure mat when the stepping pattern and weight transfer attributes could potentially be affected and measurable throughout administration of the iFSST and can underscore differences which are not perceivable with only the total duration of the FSST.

However, to analyze the FSST in a much more detailed way, especially in a cohort, adds a level of complexity for the clinical analysis and interpretation, not due to the variables themselves, but to the nature of the test and motor control strategies. The Four Square Step Test has been thought of as a simple clinical test for dynamic balance and mobility assessment, but it is also a timed test, and an agility test. Agility is the ability to quickly change speed and/or direction. As performance is increased, high agility and high balance converge, as has been shown in athletes performing a reactive agility task [29]. In this situation, some specific changes in the variables can both be considered as worsening in some individuals and as a contributing factor to the performance in others. We have mentioned previously that the limitation of the asymmetry between limbs is commonly considered as an important hallmark aspect for rehabilitation of amputee gait. Under more challenging, multi-directional tasks, and under time-pressure, the asymmetry can be an opposing feature contributing to the final performance in the most experienced transfemoral amputees, such as when, at higher levels, Paralympic amputees who ran demonstrated exacerbated inter-limb asymmetry in comparison to walking [30].

The FSST has instructions but may require further clarification to subjects. For example, it is stipulated to the individual that the feet must contact the floor in each square, but no rule defines how the foot must contact the ground. Among the participants, some will interpret it as having the full foot on the floor and others as making any contact with the floor. It is indeed possible for instance to place all the foot area on the floor or to only lightly touch the floor with a small part of the foot (most often the toes). If this second foot touches the floor completely, this implies a rebalancing of weight under the left and right support limbs. If the foot only lightly touches, then it can leave quickly to the next square, provided that the next displacement is ipsilateral. Visually, the light touch strategy gives more linear displacement of the center of pressure because the center of mass is likely swinging less, and it allows achievement of the smallest FSST duration. Also, in asymmetric populations, such as persons with a unilateral amputation, the starting limb could be fixed by the rater and the test be assessed both with a prosthetic side start and a sound limb start.

### 4.4. Limitations

A key limitation was the absence of control peers, which may have given a better relative picture of the performance and the behavior that persons with unilateral transfemoral amputation can demonstrate during the Four Square Step Test. Also, we did not consider parameters of symmetry for inclusion, for example step length and step time asymmetries during walking, which may have expanded the results. Correlation analyses between measures seems needed to determine if participants can have a deficit in one measure as a result of prioritizing improved performance in another measures. In addition, the findings are based upon the performance of individuals who may not be representative of the entire population of the persons with unilateral transfemoral amputation, as these subjects were unlimited community ambulators who lost their limbs predominantly due to trauma and malignancy. However, because current results showed discriminant features, these are highly encouraging results for future work.

## 5. Conclusions

The current study established for the first time, test-retest reliability and minimal detectable change values in adults with unilateral transfemoral amputation for the variables given by an instrumented Four Square Step Test performance, and the values for the total duration might be extended to the standard FSST, for which no minimal detectable change were available until now. Based on the decomposition of the test and information about the stepping patterns, the instrumented Four Square Step Test seems promising to allow a more comprehensive analysis beyond total time only, while it was clear that performances can be achieved using different stepping and movement strategies. It also highlighted how interpretation can be more complex when looking at the performance in a more detailed manner, where movement strategies and motor control must be considered. Overall, the results suggest this detailed assessment for the instrumented Four Square Step Test has the potential to facilitate improved rehabilitative and movement goals by considering specificities of each individual.

## Figures and Tables

**Table 1:** Demographics and characteristics for individuals with unilateral transfemoral amputation.

	TFA (*n* = 20)
Age, years, mean (SD)	46.5 (14.2)
Body Mass Index, kg/m^2^, mean (SD)	26.4 (4.2)
Gender, male, n (%)	16 (80%)
Time since amputation, years, mean (SD)	17.7 (15.6)
Amputation etiology, n (%)	
Traumatic	14 (70%)
Malignancy	4 (20%)
Peripheral vascular disease	2 (10%)
Amputation side, n (%)	
Left	7 (35%)
Right	13 (65%)
Residual limb length, %, mean (SD) (% of the sound side femur)	70 (30)
Hip flexion contracture, degrees, mean (SD)	12.8 (7.7)
Amputee Mobility Predictor, mean (SD)	40.8 (3.6)

**Table 2:** Test-retest reliability and minimal detectable change values of iFSST variables (standard left and right decomposition).

	Test 1 Mean (SD) (*n* = 40)	Test 2 Mean (SD) (*n* = 40)	Paired t-Test *p* Value	ICC2,1 (95% CI)	SEM	MDC_95_
iFSST Total Duration (s)	11.71 (3.29)	11.43 (2.98)	0.102	0.97 [0.95–0.99]	0.54	1.51 s
Sound Side Duration (s)	8.07 (3.13)	7.26 (2.91)	0.000*	0.94 [0.68–0.98]	0.74	2.05 s
Prosthetic Side Duration (s)	6.53 (2.42)	6.93 (2.25)	0.092	0.90 [0.80–0.95]	0.74	2.05 s
Duration in Transitions & Squares (% FSST)						
Tr1–2 Forward	9.71 (1.39)	10.00 (1.50)	0.040*	0.90 [0.80–0.95]	0.46	1.27%
Sq2–1	2.63 (1.26)	2.68 (1.47)	0.824	0.79 [0.59–0.89]	0.63	1.73%
Tr2–3 Right	9.57 (1.31)	9.53 (1.07)	0.764	0.84 [0.98–0.92]	0.48	1.32%
Sq3–1	2.72 (1.70)	2.66 (1.67)	0.736	0.91 [0.83–0.95]	0.51	1.40%
Tr3–4 Backward	9.66 (1.45)	9.90 (1.21)	0.233	0.73 [0.49–0.86]	0.69	1.92%
Sq4–1	4.01 (1.84)	3.42 (1.72)	0.020*	0.77 [0.54–0.88]	0.85	2.37%
Tr4–1 Left	9.52 (1.33)	9.88 (1.33)	0.075	0.73 [0.48–0.86]	0.69	1.92%
Sq1–2	3.70 (2.78)	3.14 (1.61)	0.141	0.66 [0.36–0.82]	1.28	3.55%
Tr1–4 Right	9.88 (1.71)	9.90 (1.37)	0.926	0.88 [0.77–0.94]	0.53	1.48%
Sq4–2	2.57 (1.28)	2.71 (1.81)	0.444	0.85 [0.72–0.92]	0.60	1.66%
Tr4–3 Forward	9.56 (1.33)	9.69 (1.26)	0.275	0.90 [0.82–0.95]	0.41	1.14%
Sq3–2	3.41 (1.85)	3.32 (1.67)	0.724	0.74 [0.50–0.87]	0.90	2.49%
Tr3–2 Left	9.54 (1.27)	9.80 (1.34)	0.121	0.82 [0.65–0.90]	0.55	1.53%
Sq2–2	3.42 (2.38)	3.04 (1.67)	0.102	0.86 [0.73–0.93]	0.76	2.10%
Tr2–1 Backward	10.10 (1.50)	10.32 (1.32)	0.157	0.87 [0.75–0.93]	0.64	1.78%
Stepping Pattern						
Extra Steps Taken (n)	2.24 (2.50)	1.79 (2.00)	0.030*	0.91 [0.83–0.96]	0.68	1.87 steps
Changes of Main Support (n)	17.50 (3.49)	16.95 (3.66)	0.096	0.91 [0.83–0.95]	1.07	2.97 changes
COP Pathway’s Efficiency (%)	50.6 (6.2)	51.0 (6.1)	0.496	0.92 [0.84–0.96]	1.74	4.8%

iFSST is instrumented Four Square Step Test. Tr is transition. Sq is square. COP is Center of Pressure. * Paired t-test *p*-value < 0.05, which suggests true difference between Test 1 and Test 2 means.

**Table 3:** Test-retest reliability and minimal detectable change values of iFSST variables (decomposition based on prosthetic side and sound side).

	Test 1 Mean (SD) (*n* = 40)	Test 2 Mean (SD) (*n* = 40)	Paired t-Test *p* Value	ICC2, 1 (95% CI)	SEM	MDC_95_
Lateral Transition toward prosthetics side						
Swing phase duration (s)	0.49 (0.09)	0.50 (0.10)	0.891	0.68 [0.46–0.82]	0.05	0.15 s
Step width (cm)	72.9 (6.6)	72.0 (6.4)	0.275	0.73 [0.54–0.85]	3.4	9.5 cm
Lateral Transition toward sound side						
Swing phase duration (s)	0.72 (0.14)	0.69 (0.15)	0.202	0.43 [0.14–0.65]	0.11	0.29 s
Step width (cm)	75.9 (6.5)	74.8 (7.6)	0.046*	0.89 [0.79–0.94]	2.3	6.5 cm
Asym. Ratio (toward prosth/toward sound)						
Swing phase duration	0.71 (0.17)	0.74 (0.13)	0.316	0.39 [0.08–0.63]	0.12	0.32
Step width	0.96 (0.06)	0.96 (0.05)	0.781	−0.02 [−0.34–0.31]	0.06	0.15

* Paired t-test *p*-value < 0.05, which suggests true difference between Test 1 and Test 2 means.

**Table 4:** Detailed assessments for the iFSST in persons with TFA with the C-Leg or the Genium MPK (standard left and right decomposition). Arrows in the last column indicate the direction of the change (increase or decrease) for variables with significant difference.

	PwTFA with C-Leg MPK (*n* = 16)		PwTFA with Genium MPK (*n* = 16)		CLeg versus Genium	
	Mean (SD)	[95% CI]	Mean (SD)	[95% CI]	*p* =	
iFSST Total Duration (s)	11.09 (1.93)	[10.06–12.11]	10.36 (2.75)	[8.89–11.82]	0.090	
Sound Side Duration (s)	7.35 (2.04)	[6.27–8.44]	6.44 (2.51)	[5.10–7.77]	**0.036**	↓
Sound Side Duration (% FSST time)	65.5 (8.7)	[60.9–70.2]	60.8 (10.1)	[55.4–66.2]	**0.033**	↓
Prosthetic Side Duration (s)	6.40 (1.56)	[5.57–7.23]	6.20 (2.35)	[4.94–7.45]	0.558	
Prosthetic Side Duration (% FSST time)	57.3 (6.9)	[53.6–61.0]	58.8 (9.7)	[53.6–64.0]	0.502	
Starting by Prosthetic or Sound Side (n/n)	11/5		11/5			
Duration in Transitions & Squares (% FSST)						
Tr1–2 Forward	9.4 (1.4)	[8.6–10.1]	10.3 (1.7)	[9.5–11.2]	****0.001****	↑
Sq2–1	2.5 (1.2)	[1.8–3.1]	2.3 (1.3)	[1.6–3.0]	0.132	
Tr2–3 Right	9.4 (0.9)	[9.0–9.9]	9.9 (1.0)	[9.4–10.5]	0.139	
Sq3–1	2.6 (1.9)	[1.6–3.6]	2.2 (1.4)	[1.5–3.0]	0.250	
Tr3–4 Backward	10.0 (1.3)	[9.3–10.7]	9.7 (1.3)	[9.0–10.4]	0.244	
Sq4–1	3.8 (1.2)	[3.2–4.5]	3.1 (1.7)	[2.2–4.1]	**0.049**	↓
Tr4–1 Left	9.4 (1.1)	[8.8–9.9]	10.2 (1.0)	[9.6–10.7]	**0.005**	↑
Sq1–2	4.0 (3.3)	[2.2–5.7]	2.6 (1.4)	[1.9–3.4]	**0.048**	↓
Tr1–4 Right	9.9 (1.2)	[9.3–10.6]	10.3 (1.5)	[9.5–11.1]	0.162	
Sq4–2	2.3 (0.9)	[1.8–2.8]	2.4 (1.4)	[1.6–3.2]	0.808	
Tr4–3 Forward	9.4 (1.1)	[8.8–10.0]	10.3 (1.4)	[9.6–11.1]	**0.003**	↑
Sq3–2	3.3 (2.2)	[2.2–4.5]	3.3 (1.9)	[2.3–4.3]	0.910	
Tr3–2 Left	9.8 (1.0)	[9.2–10.3]	10.4 (1.3)	[9.7–11.1]	**0.018**	↑
Sq2–2	3.2 (1.6)	[2.4–4.0]	2.8 (1.9)	[1.7–3.8]	0.263	
Tr2–1 Backward	10.9 (1.0)	[1.04–11.5]	10.1 (1.3)	[9.4–10.8]	0.055	
Transitions/Squares Duration Ratio	4.0 (1.6)	[3.2–4.9]	5.2 (2.4)	[3.9–6.5]	**0.003**	↑
Stepping Pattern						
Extra Steps Taken (n)	2.25 (1.69)	[1.35–3.15]	1.38 (1.63)	[0.51–2.24]	**0.014**	↓
Changes of Main Support (n)	17.25 (2.02)	[16.18–18.32]	15.75 (3.66)	[13.80–17.70]	**0.029**	↓
COP Pathway’s Efficiency (%)	51.4 (5.0)	[48.7–54.0]	52.7 (5.2)	[49.9–55.5]	0.168	

Bold values denote statistical significance at the *p* < 0.05 level.

**Table 5:** Detailed assessments for the iFSST in persons with TFA with the C-Leg or the Genium MPK (decomposition based on prosthetic side and sound side).

	PwTFA with C-Leg MPK (*n* = 16)		PwTFA with Genium MPK (*n* = 16)		CLeg versus Genium	
	Mean (SD)	[95% CI]	Mean (SD)	[95% CI]	*p* =	
Lateral Transition toward prosthetic side						
Swing phase duration (s)	0.51 (0.09)	[0.47–0.56]	0.50 (0.10)	[0.45–0.55]	0.341	
Step width (cm)	71.7 (6.0)	[68.5–74.9]	72.4 (6.2)	[69.1–75.7]	0.705	
Lateral Transition toward sound side						
Swing phase duration (s)	0.68 (0.11)	[0.63–0.74]	0.71 (0.17)	[0.63–0.80]	0.498	
Step width (cm)	74.8 (6.8)	[71.1–78.4]	76.0 (7.3)	[72.1–79.8]	0.448	
Asym. Ratio (toward prosth/toward sound)						
Swing phase duration	0.76 (0.11)	[0.70–0.81]	0.72 (0.18)	[0.63–0.82]	0.523	
Step width	0.96 (0.05)	[0.93–0.99]	0.95 (0.06)	[0.92–0.99]	0.769	

## Supplementary Materials

The following are available online at https://www.mdpi.com/1424-8220/20/17/4782/s1, Video S1: Video of two FSST performed in the same range of time, but in different ways.
